# 

**Published:** 1898-06-04

**Authors:** 


					TH E HUH FIT A U. " ine sianaarci 01 nignesi puiuy. ?mumi. ^
CADBURY'S WW ^ CADBURY'S
C/ COCOA ?'111 iPl J?. COCOA
ABSOLUTELY PURE. JBt) s=? <=> i NO DRUGS OR ALKALIES USED
'/? WICH HOSPITAL
No. 610. Vol. XXIV. CfSSSSa Satubday,
June 4th, 1898.
[BnbsonpUon Torras.j pRICE TWOFENCF.

				

